# A Tetrahedral Silver-Rich Supercluster Composed of 8‑Electron IrH_2_Ag_12_ Icosahedra

**DOI:** 10.1021/jacs.5c22409

**Published:** 2026-05-29

**Authors:** Tzu-Hao Chiu, Michael N. Pillay, Yoshiki Niihori, Yuichi Negishi, Samia Kahlal, Jean-Yves Saillard, C. W. Liu

**Affiliations:** † Department of Chemistry, 63373 National Dong Hwa University, Hualien 97401, Taiwan (Republic of China); ‡ Institute of Multidisciplinary Research for Advanced Materials, 13101 Tohoku University, Katahira 2-1-1, Aoba-ku, Sendai 980-8577, Japan; § Univ Rennes, CNRS, ISCR-UMR 6226, F-35000 Rennes, France

## Abstract

Although the prediction of constructing three-dimensional self-assembled superclusters using icosahedral superatoms as building blocks dates back to 1987, this task remains a significant challenge, due to the difficulty in synthesizing and characterizing such clusters. In this study, the 8-electron superatom IrH_2_Ag_19_[S_2_P­(O^*n*^Pr)_2_]_12_ (**1**) was discovered for the first time, with IrH_2_ as the dopant. Its distinctive superatomic core, IrH_2_Ag_12_, was utilized as a building block for the self-assembly of a tetrahedral supercluster. A new hydride-containing 32-electron Ir/Ag alloy nanocluster, (IrH_2_)_4_Ag_50_[S_2_P­(O^*n*^Pr)_2_]_22_ (**2**), was prepared using homoleptic ligand. Similar to the prediction by Teo et al., the four icosahedra are assembled in a tetrahedral manner, which is reminiscent of tetrahedrane, to form the [(IrH_2_)_4_Ag_42_]^14+^ core passivated by [Ag_8_{S_2_P­(O^*n*^Pr)_2_}_22_]^14–^. Unlike previous studies that used M_13_ units, this study uniquely utilizes four (IrH_2_)@Ag_12_ units sharing six vertices to achieve the tetra-icosahedral kernel. The discovery of this 3D supercluster can more effectively help us understanding the growing process from nanoclusters to nanoparticles.

## Graphical abstract

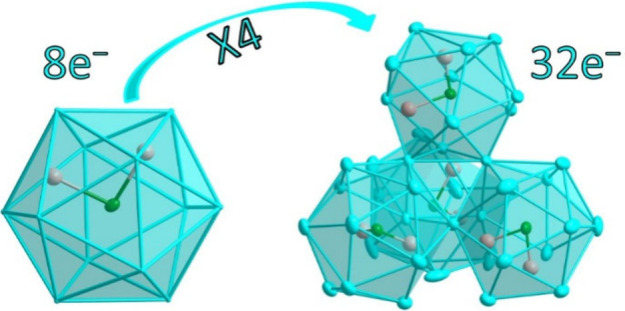

## Introduction

Since the beginning of this century, atomically precise nanoclusters (NCs), which fall in size between atoms and nanoparticles (NPs), have attracted increasing attention due to their well-defined unique compositions and structures, as well as their distinct optical properties, making them promising materials for various applications.^1–9^ In the field of group 11 metal nanoclusters, doping with metals from other groups to enhance stability or modify properties has been practiced for many years.^9–15^ More recently, it has been discovered that using nongroup 11 metals as dopants can potentially be accompanied by hydrides codoping, when borohydride is used as a reducing agent in the one-pot synthesis of group 11 superatomic NCs.^16–28^ In such cases the hydride codopant is encapsulated within the NC superatomic core. When doped with group 10 metals, a single hydride can be inserted into the superatom, as seen in [MHCu_11_(dtp)_6_(CCPh)_4_] (M = Pd,^16^ Pt;^17^ dtp = S_2_P­(OR)_2_), [PtHAg_19_(dtp/desp)_12_] (dsep = Se_2_P­(OR)_2_),^18^ [PdHAg_19_(dtp/desp)_12_],^19,20^ [PdHAg_20_(dsep)_12_]^+^,^20^ [NiHAg_19_(dtp)_12_][Bibr ref21] and [PtHPtAg_32_(dtp)_17_].^22^ When the dopants are group 9 or group 8 metals, up to two hydrides can be introduced into the superatom, as observed in [RhH_*x*_@Ag_21‑x_(dtp/dsep)_12_] (x = 1, 2),^23,24^ [(MH)­Ag_24_(SR)_18_]^2–^ (M = Rh, Ir),^25,26^ [(MH_2_)­Ag_24_(SR)_18_]^2–^ (M = Ru, Os).^26^ A copper-rich trihydride NC has also been reported.^27^ Such codopant hydrides differ from traditional hydride ligands as their electrons participate in the total superatomic electron count of the NC.

The concept of using M_13_-centered icosahedra as building blocks to form 3D superclusters (or clusters of clusters) via vertex-sharing strategies was first proposed in the eighties by Teo et al.^29,30^ Because of the challenges in synthesis, there are only a few examples of ligand-protected supercluster cores composed of more than two centered icosahedra. Tri-icosahedral core structures include the linear M_37_ arrangement (Scheme 1a), present in [Au_37_(PPh_3_)_10_(SC_2_H_4_Ph)_10_X_2_]^+^ (X = Cl, Br)^31^ and Pt_3_Ag_44_(dtp)_22_ ^13^ and the cyclic M_36_ arrangement (Scheme 1b) existing in [Pt_3_Ag_33_(PPh_3_)_12_Cl_8_]^+^ cores.^14^ Linear M_49_ and cyclic M_48_ tetra-icosahedral architectures (Scheme 1c,d) have also been reported as constituting the cores of larger NCs, as exemplified by Ag_61_(dpa)_27_ (Hdpa = dipyridylamine)^32^ and Au_52_(HOPPh_2_)_8_(OPPh_2_)_4_(SR)_16_.^33^ Finally, the largest cluster of clusters structure reported so far is the M_60_ cyclic penta-icosahedral arrangement (Scheme 1e), which constitutes the core of [Au_60_Se_2_(Ph_3_P)_10_(SePh)_15_]^+^.^34^ The aforementioned examples exhibit linear or planar arrangements of icosahedra, with the exception of the puckered ring formed by the tetraicosahedral Au_52_(HOPPh_2_)_8_(OPPh_2_)_4_(SR)_16_ (Scheme 1d). It is of note that this geometry differs from the M_46_ tetra-icosahedral tetrahedral arrangement originally predicted.^29,30^ Herein, we report the synthesis and characterization of two novel Ag-rich NCs codoped with IrH_2_ motifs encapsulated within metallic icosahedral cages, namely, the 8-electron [IrH_2_Ag_19_[S_2_P­(OPr)_2_]_12_ (**1**) and the 32-electron (IrH_2_)_4_Ag_50_[S_2_P­(OPr)_2_]_22_ (**2**). The latter NC features an unprecedented tetrahedral arrangement of four (IrH_2_)@Ag_12_ icosahedra sharing six vertices.^29,30^ While the S_2_P­(OR)_2_ ligand is known to stabilize a variety of nanocluster morphologies,^35^ the emergence of this specific tetrahedral assembly is primarily driven by the central Ir dopant. We propose that the Ir–H core induces a specific deformation of the icosahedral subunits, creating a geometric template that favors tetrahedral packing over previously reported architectures.

**Scheme 1:**
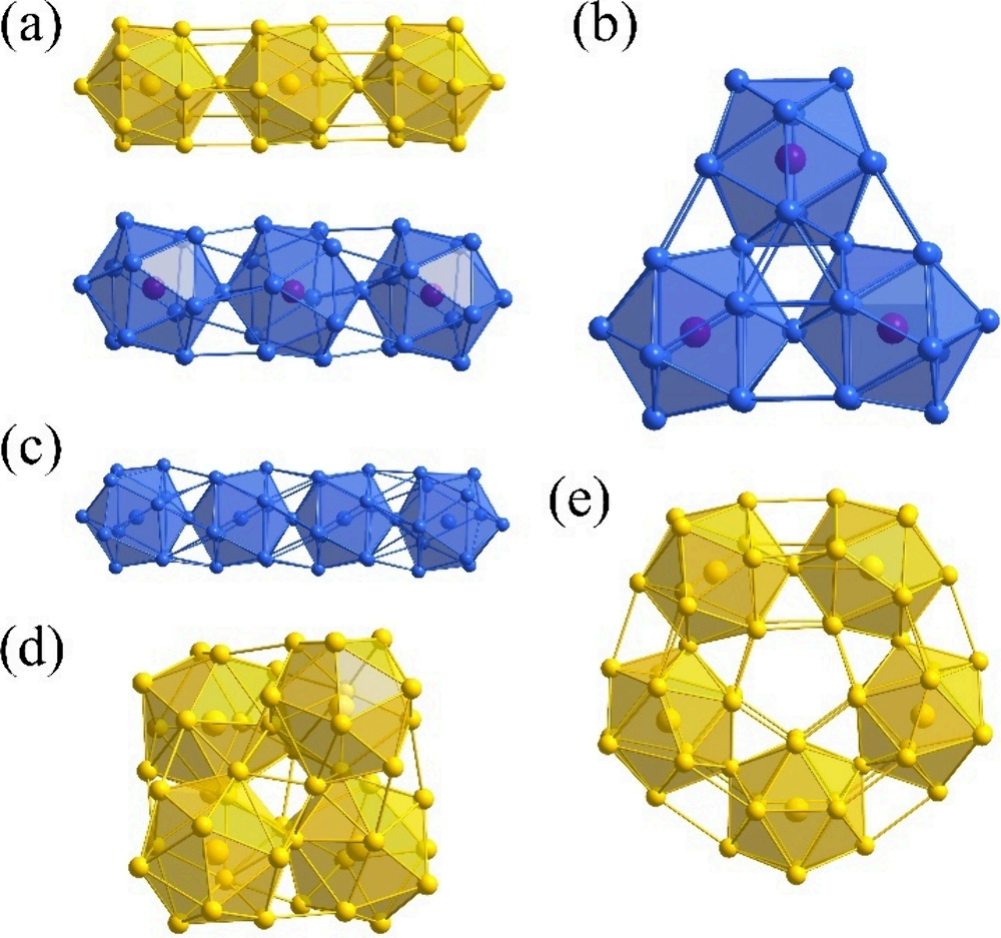
(a) Linear tri-icosahedral Core of [Au_37_(PPh_3_)_10_(SC_2_H_4_Ph)_10_X_2_]^+^ (X = Cl, Br) and Pt_3_Ag_44_(dtp)_22_; (b) Cyclic Tri-icosahedral Core of [Pt_3_Ag_33_(PPh_3_)_12_Cl_8_]^+^; (c) Linear Tetra-icosahedral Core of Ag_61_(dpa)_27_; (d) Cyclic Tetra-icosahedral Core of Au_52_(HOPPh_2_)_8_(OPPh_2_)_4_(SR)_16_; (e) Cyclic Penta-icosahedral Core of [Au_60_Se_2_(Ph_3_P)_10_(SePh)_15_]^+^ ^a^

a Color code: gold for Au; blue for Ag; purple for Pt.

## Results and Discussion

Compounds **1** and **2** were prepared by a one-pot reaction method. The reaction involves a mixture of Ag­(MeCN)_4_PF_6_, NH_4_[S_2_P­(OPr)_2_], [Ir­(COD)­Cl]_2_, and LiBH_4_ in a molar ratio of 13:7:1:13, respectively, with THF as the solvent. The reaction was carried out at 253 K for 12 h. The reaction mixture was further purified by using thin-layer chromatography (TLC). Dark red block crystals of **1** were grown from a MeOH/hexane solution over 1 week at room temperature. Compound **2** was crystallized similarly but by using pure MeOH as the solvent.

The solid-state structures of **1** and **2** were elucidated by the single-crystal X-ray diffraction. In the absence of neutron diffraction data, the hydride positions were assigned on the basis of DFT calculations and comparison with previously reported Rh-doped analogues.^23^ Compound **1** crystallizes in a monoclinic space group and, similarly to [RhH_2_Ag_19_(dtp)_12_],^23^ includes cocrystallized hexane molecules. The metal framework of compound **1** consists of a distorted centered icosahedral (IrH_2_)@Ag_12_ core, surrounded by a protecting shell composed of 7 silver capping atoms and 12 dtp ligands (Figure 1a). This unsymmetrical outer shell is similar to those in the previously reported 8-electron species of the type [M*Ag_19_(dtp)_12_] (M* = Ag, Au, PdH, PtH, RhH_2_),^18,19,23,36,37^ reducing the ideal symmetry of the whole cluster to *C*_1_. The hydride positions were determined by residual electron density and refined with constraints.^38^ The two hydrides in **1** exhibit distinct coordination modes: one tricoordinated (to Ir and 2 Ag atoms) and the other tetracoordinated (to Ir and 3 Ag atoms), with an H–Ir–H angle of 94°. This is a relatively similar result to that observed in the neutron-determined structure of the rhodium analogue, [RhH_2_@Ag_19_(dtp)_12_].^23^ A slight difference is that the icosahedron in compound **1** exhibits disordered silver atoms (Figure S1a) which is likely caused by hydride migration.^39^ Notably, **1** is the first reported icosahedral NC centered by an IrH_2_ unit. The presence of two hydrides weakens the Ir–Ag interactions, resulting in an average Ir–Ag distance in **1** (2.82 Å) that is longer than in the monohydride [(IrH)­Ag_24_(SR)_18_]^2–^ (2.78 Å).^26^ Furthermore, the distortion of the icosahedron is larger, as indicated by their respective values of continuous symmetry measure (CSM)^40^ value of **1** (0.43) compared to [(IrH)­Ag_24_(SR)_18_]^2–^ (0.35).

**Figure 1:**
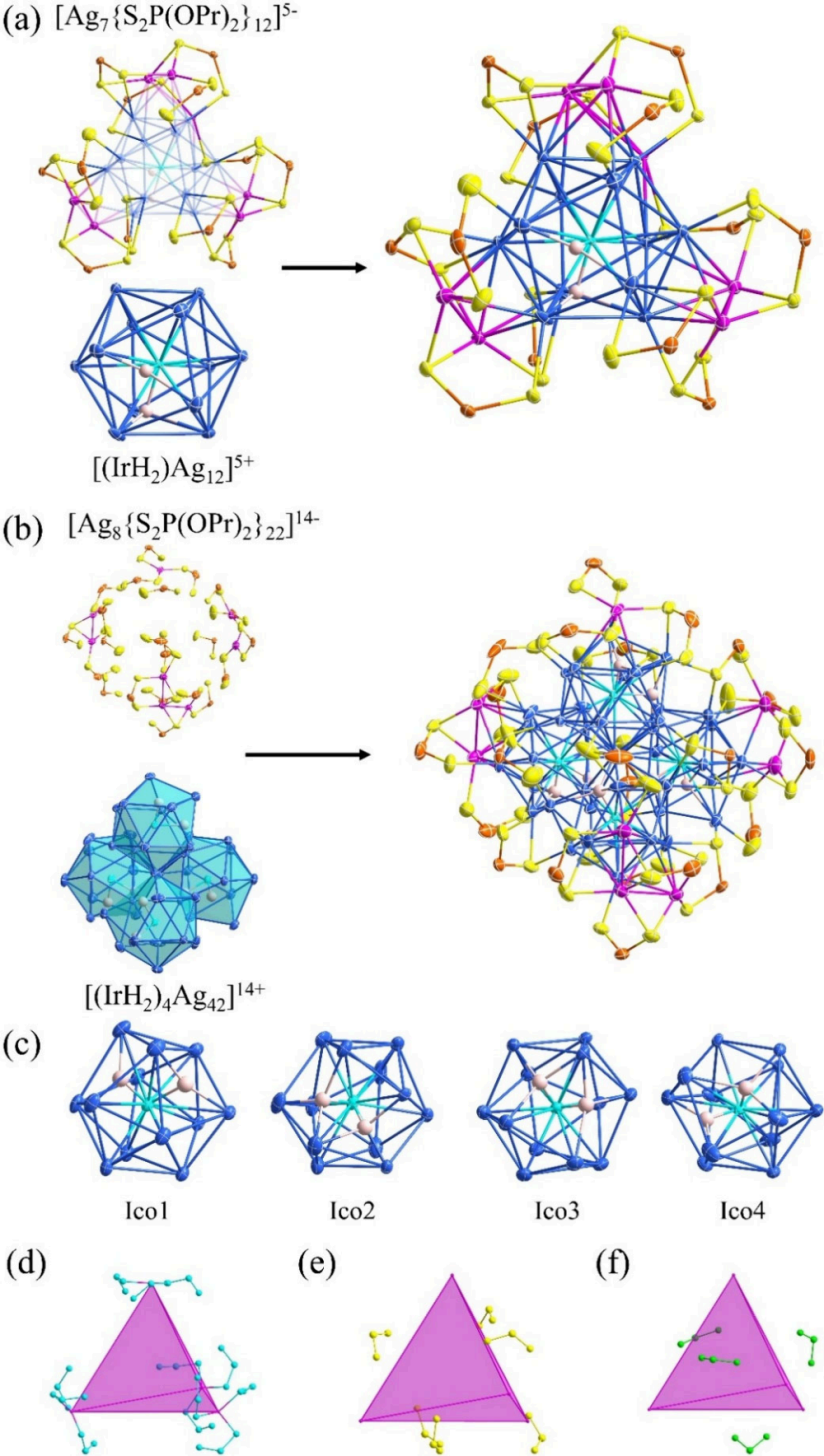
(a) IrH_2_@Ag_12_ core and passivation layer of **1**. (b) Tetra-icosahedral core and passivation layer of **2**. (c) Four different distorted icosahedral core IrH_2_@Ag_12_ of **2**. (d, e, f) Three ligand groups in **2**. Color code: yellow for S, orange for P, magenta for Ag_cap_, blue for Ag_ico_, sky blue for Ir, pink for H.

The (IrH_2_)_4_Ag_42_ core of **2** consists of the assembly of four distorted (IrH_2_)@Ag_12_ centered icosahedra. Each icosahedron shares one vertex with each of the other three, resulting in a total of six shared vertices (thus 4 × 12 – 6 = 42 Ag atoms). The shared vertices form an octahedron, and four of its tetrahedrally arranged faces are each shared with a face of one of the four icosahedra, positioning the centers of the four icosahedra to form a tetrahedron. Although a regular arrangement of the icosahedra would yield ideal *T*_d_ symmetry, the distortions caused by the encapsulated hydrides result in the (IrH_2_)_4_Ag_42_ core of **2** exhibiting *C*_1_ symmetry. Since the four icosahedra are somewhat different, they are individually designated as Ico1, Ico2, Ico3, and Ico4 for detailed analysis (Figure 1c). The (IrH_2_)_4_Ag_42_ core is protected by an outer passivation layer made of 8 peripheral Ag atoms and 22 dtp ligands (Figure 1b). The eight outer Ag atoms divide into four groups in 1:2:2:3 ratios, capping either edges or triangular faces of the four icosahedra, respectively. The CSM values for the four icosahedral units comprising the (IrH_2_)_4_Ag_42_ inner core, which vary between 1.10 and 1.49 (Table 1), are fairly large indicating substantially distorted icosahedra. Analyzing these distortions allowed us to locate the hydrides in the vicinity of the enlarged triangular faces or edges. The Ir–Ag distance is in the range 2.716–3.096 Å with an average of 2.834 Å which is slightly longer than that in **1**. The metal framework in compound **2** also exhibits Ag_ico_ disorder, which can be attributed to hydride migration (Figure S1b). Specifically, in Ico3, a Ag_3_ unit is disordered into two positions with occupancies of 90% and 10% (Figure S2). More complicated disorders were found in Ico2 and Ico4, where each of them can be separated into three different icosahedral units with occupancies of 10%, 35%, and 55%, respectively (Figures S3–S4).

**Table 1:** Selected Experimental and Computed Distances (Å) and Angles (deg) for **1** and **2**

		**2**			
**Compd**	**1**	**Ico1**	**Ico2**	**Ico3**	**Ico4**
**SCXRD**					
**CSM**	0.43	1.1	1.07	1.46	1.49
**Ir–Ag**_**ico**_	2.763(1)–2.936(1)	2.716(2)–3.010(2)	2.736(2)–3.008(2)	2.741(2)–3.096(2)	2.730(2)–3.050(3)
	avg. 2.820(1)	avg. 2.840(2)	avg. 2.831(2)	avg. 2.849(2)	avg. 2.835(2)
**Ag**_**ico**_ **–Ag**_**ico**_	2.789(1)–3.694(2)	2.730(2)–3.932(2)	2.663(4)–3.877(2)	2.702(2)–4.125(2)	2.507(5)–3.924(4)
	avg. 2.964(2)	avg. 2.995(2)	avg. 2.960(3)	avg. 2.964(2)	avg. 2.996(4)
**Ag**_**ico**_ **–Ag**_**cap**_	2.922(1)–3.132(1)	2.862(2)–3.087(2)			
	avg. 3.021(1)	avg. 2.968(2)			
**DFT**					
**CSM**	0.69	1.03	1.06	1.23	0.99
**Ir–Ag**_**ico**_	2.866–3.007	2.794–3.109	2.815–3.064	2.850–3.162	2.812–3.129
	avg. 2.926 [0.151]	avg. 2.933 [0.149]	avg. 2.946 [0.139]	avg. 2.943 [0.145]	avg. 2.934 [0.138]
**Ag**_**ico**_ **–Ag**_**ico**_	2.900–3.844	2.882–4.045	2.865–4.006	2.850–4.210	2.835–4.002
	avg. 3.078 [0.062]	avg. 3.091 [0.056]	avg. 3.074 [0.060]	avg. 3.092 [0.061]	avg. 3.089 [0.058]
**Ag**_**ico**_ **–Ag**_**cap**_	3.035–3.231	2.991–3.230			
	avg. 3.136 [0.036]	avg. 3.097 [0.042]			
**Ir–H**	1.677–1.687	1.672–1.680	1.662–1.677	1.655–1.656	1.665–1.683
	avg. 1.682 [0.288]	avg. 1.676 [0.285]	avg. 1.669 [0.308]	avg. 1.656 [0.328]	avg. 1.674 [0.297]
**H–Ir–H**	100	90	85	79	90

The four central Ir atoms describe a large tetrahedron with Ir. . . Ir distances ranging from 5.622 to 5.925 Å, indicating that the four icosahedra are assembled in a quite regular tetrahedral way (Figure S5). The 22 ligands in **2** can be divided into three groups in a 6:3:2 ratio. The first group, comprising 12 ligands, is evenly distributed into four sets, each surrounding one of the four vertices of the large tetrahedron (Figure 1d). The second group, containing 6 ligands, bridges the six edges of the large tetrahedron (Figure 1e) while the final four ligands cap its four large triangular faces (Figure 1f). The compositions of **1** and **2** were determined by positive ESI-MS spectroscopy. The most intense peak in compound **1** corresponds to the [**1** + Ag]^+^ adduct which showed good alignment with the simulated spectrum (Figure 2a) ([**1** + Ag]^+^: Exp.: 4911.4604 Da; Calc.: 4911.2827 Da). The signals of **2** were observed around 5500 and 3600 Da. The dicationic adducts [**2** + 2Ag]^2+^ and [**2** + 2H]^2+^ were observed around 5500 Da ([**2** + 2Ag]^2+^: Exp: 5538.7024, Calc: 5538.6659; [**2** + 2H]^2+^: Exp: 5431.8376, Calc: 5431.7731). The tricationic adduct [**2** + Ag + 2H]^3+^ was observed at 3657.4544. (Calc. 3657.4838) (Figure 2b). In addition, the ESI-MS spectra of the deuterated analogues **1**_**D**_ and **2**_**D**_ exhibit slightly different features from those of their hydride counterparts. In the spectrum of **1**_**D**_, although the signal corresponding to [**1**_**D**_ + Ag]^+^ was not observed, a peak assignable to [**1_D_** + H]^+^ was detected, showing a mass shift of 2 Da relative to the corresponding hydride species, consistent with deuterium incorporation. In the spectrum of **2**_**D**_, peaks were observed that are shifted by 4 Da relative to the [**2** + 2H]^2+^ and [**2** + 2Ag]^2+^ ions, respectively (Figures 2c, S6).

**Figure 2:**
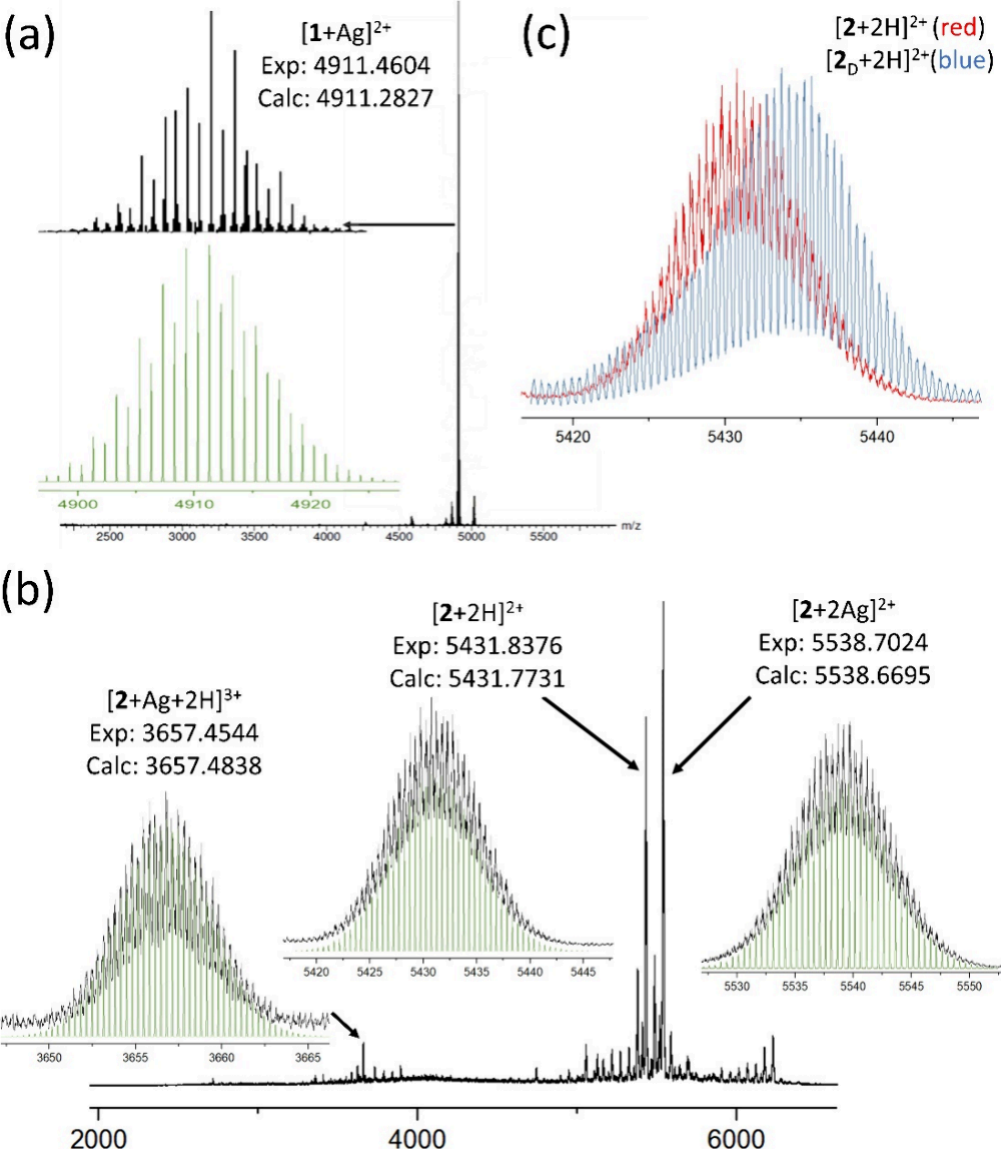
Positive-mode ESI mass spectrum of (a) **1**. Insets: experimental (black) and simulated (green) isotopic distribution of the ion peak [**1**+Ag]^+^. (b) **2**. Insets: experimental (black) and simulated (green) isotopic distribution of the ion peak [**2** + Ag + 2H]^3+^, [**2** + 2H]^2+^, [**2** + 2Ag]^2+^. (c) Isotopic distribution of the ion peak [**2** + 2H]^2+^ and [**2**_**D**_ + 2H]^2+^.

The absorption and photoluminescent (PL) spectra of **1** and **2** in 2Me-THF are shown in Figure 3. Compared to RhH_2_Ag_19_, a slightly red-shifted absorption band can be observed in the absorption spectrum of compound **1** at 410 nm. The absorption spectrum of **2** shows a broad band at 400 nm. Unlike one-dimensional (1D) metal nanostructures, which typically exhibit absorption shifts toward the near-infra region due to cluster growth along a single direction, compound **2** lacks a significant aspect ratio. The PL of **1** at 77 K is located near 720 nm. In contrast, the PL of **2** exhibits a red shift to 847 nm. (lifetime of **1**:39 μs; **2**:178 μs; Figure S7). The PLQY of compounds **1** and **2** at 77 K are 39.1% and 8.0%, respectively (**1**: *k*_r_ = 1.0 × 10^4^ s^–1^, *k*_nr_ = 1.56 × 10^4^ s^–1^; **2**: *k*_r_ = 4.2 × 10^2^ s^–1^, *k*_nr_ = 5.2 × 10^3^ s^–1^). Combined with their lifetimes, the derived rate constants indicate that the substantial decrease in the *k*_r_ of compound **2** predominantly accounts for its lower quantum yield. This behavior is consistent with the reduced spatial overlap of the frontier orbitals in compound **2**, which would diminish the transition dipole moment and oscillator strength.

**Figure 3:**
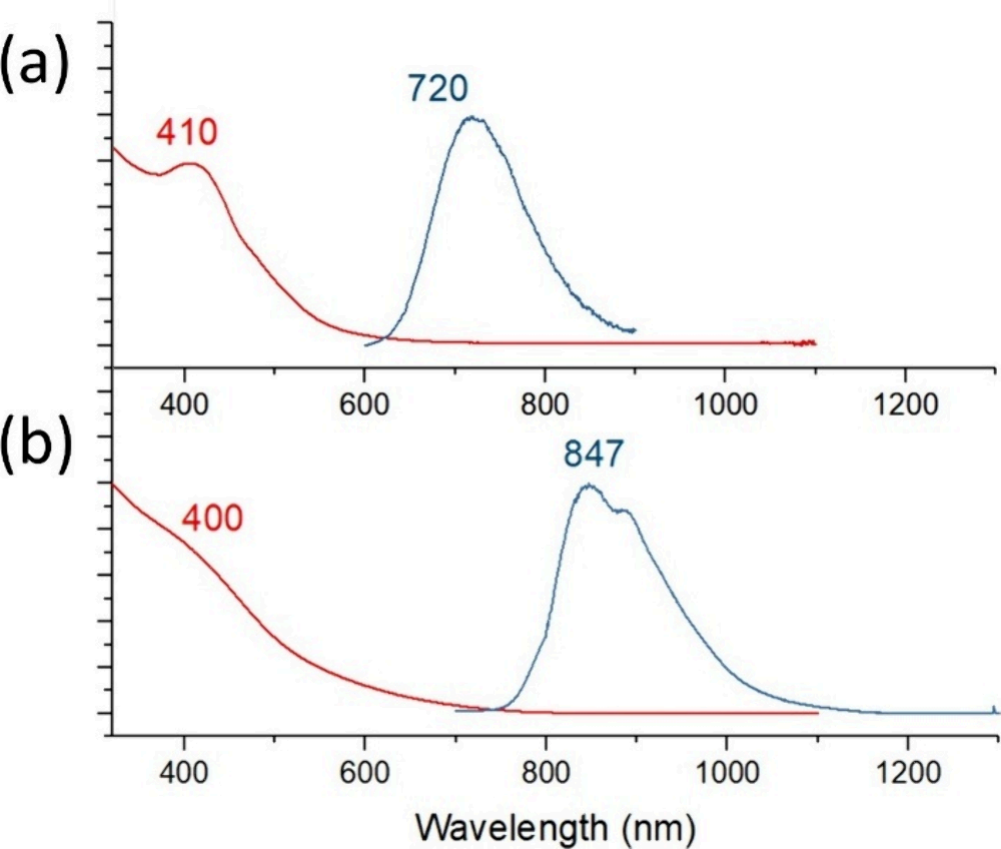
Absorption and 77 K photoluminescence spectra of **1** and **2**.

The ^31^P NMR spectrum of **1** shows one sharp peak at 105.0 ppm, and that of **2** shows two peaks around 105.3 and 98.7 ppm. The ^1^H NMR spectrum of **1** shows a sharp resonance for the hydride centered at −17.6 ppm, with an integration ratio of 1:6 between the hydride and the ligand. The ^1^H NMR spectrum of **2** displays a single hydride peak at −14 ppm, with an integration ratio of 4:11 between the hydride and the ligands. The ^2^H NMR spectrum of the deuteride species **1** provides additional support for the hydride signal assignment. The downfield chemical shift of the hydrides indicates that a looser metal framework exhibits a weaker shielding effect (Figures S8–S12).

Based on their compositions, and assuming that the 11-electron IrH_2_ dopant is isoelectronic to the 1-electron Au or Ag atoms, **1** and **2** have 8 and 32 electrons, respectively (**1**: 1 + 19 – 12 = 8; **2**: 1 × 4 + 50 – 22 = 32). In that way, **2** is similar to most superclusters composed of vertex-sharing centered icosahedral units, with a closed-shell count of 8 electrons assigned to each unit, thus 8 × 4 = 32 electrons. In order to get a deeper insight into the bonding in **1** and **2**, DFT calculations at the BP86/Def2-TZVP level were performed on both NCs (see Computational Details in the SI). For the sake of computational limitations, the O^*n*^Pr groups on the ligands were replaced by hydrogen atoms, a simplification that has been proven to be satisfying in many previous investigations.^16–24,36,37,41–43^ Selected computed data are given in Tables 1 and 2. The optimized geometries of **1** and **2** are in a rather good agreement with their experimental X-ray counterparts (Table 1) including for the hydride positions. The computed H–Ir–H angles of **2** lie in the narrow range 79°–90°, whereas that in **1** is slightly larger (100°). The Ir–H distances ranging 1.66–1.68 Å indicate strong bonding between the codopants. The average atomic charges calculated from a natural population analysis (NPA) are consistent with the fact that the cores of **1** and **2** are made up of individual [IrH_2_@Ag_12_]^5+^ icosahedral units, one isolated in **1** and four quasi-independent sharing vertices in **2**. They are also consistent with the fact that the capping Ag atoms are in their +I oxidation state. In fact, these charges are quite similar to those found previously for the rhodium analogue of **1**,^23^ and many other related 8-electron systems.^13,18–24,36,37,41^ This is also supported by the Kohn–Sham orbital diagrams of **1** and **2** (Figures 4, S13). The three highest occupied orbitals of **1** can be identified as the 1P *superatomic* orbitals, and their five lowest vacant ones, as the 1D levels. In the case of **2**, there are 12 occupied combinations of the 1P orbitals and 20 combinations of the 1D orbitals. The former constitute the highest occupied block, somewhat mixed with 5d­(Ir) orbitals, and the latter are vacant, participating to the lowest unoccupied orbitals. Owing to the (weak) interaction between its four icosahedral building blocks, the HOMO–LUMO gap of **2** (1.23 eV) is reduced comparatively with that of **1** (1.89 eV). As found for their various hydride-containing rhodium relatives,^23^ the participation of the 1s­(H) orbital into the 1P and 1D *superatomic* orbitals in **1** and **2** is small (Figure 4).

**Figure 4:**
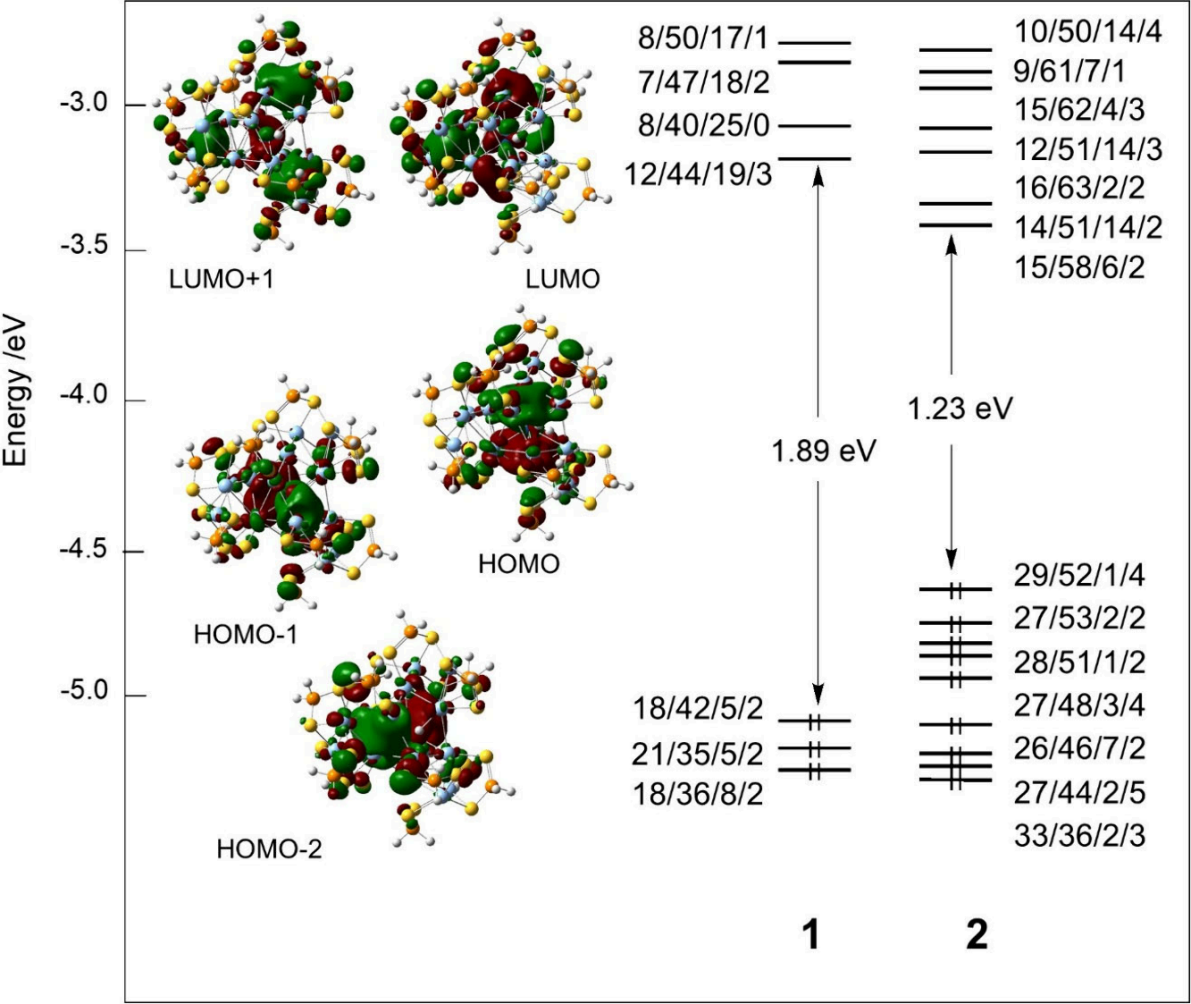
Kohn–Sham frontier orbital diagrams of **1** and **2**. Atomic contributions (in%) are given in the order: Ir/Ag_ico_/Ag_cap_/H.

**Table 2:** Averaged Atomic Charges Computed from Natural Population Analyses for **1** and **2**

**Averaged NPA charges**	**1**	2
**Ir**	–1.16	–1.13
**Ag**_**ico**_ **(Ag**_**shared**_ **)**	0.31	0.30 (0.11)
**Ag**_**cap**_	0.57	0.58
**H**	–0.28	–0.25

TD-DFT calculations at the CAM-B3LYP/Def2-TZVP level (see Computational Details in the SI) were performed on **1** and **2** to understand their UV–vis absorption behavior. The simulated spectra (Figure S14) are in a satisfying agreement with their experimental counterparts, and the low-energy band in **1** is situated at 362 nm. It is of dominant 1P → 1D character. The featureless spectrum of **2** can be interpreted as resulting from the addition of the spectra of four weakly interacting but slightly different IrH_2_@Ag_19_ icosahedral units, resulting in substantial band broadening, from which the shoulder that emerges at ∼430 nm is the counterpart of the low-energy band of **1**, with similar character.

## Conclusion

We report the first IrH_2_-doped silver-rich superatom and supercluster, whose cores contain distorted icosahedral IrH_2_@Ag_12_ unit(s). Compound **1** represents a new addition to the family of the 8-electron silver-rich superatomic clusters. Compound **2** features a self-assembled structure of four-centered icosahedra, a formation predicted as early as 1987.^29^ Owing to the absence of a pronounced aspect ratio, compound **2** exhibits an absorption spectrum that differs from those of 1D-grown clusters, particularly in the NIR region. Although each icosahedral core contains eight electrons, meaning that there is no significant interaction between the centered icosahedral units, this rare 3D homoleptic, silver-rich supercluster, formally equivalent to a Ne_4_ van der Waals tetramer, opens a new chapter in cluster chemistry. Furthermore, the exploration of different dopants and their effects on the stability and properties of these NCs continue to be a critical area of research. The unique optical and electronic properties arising from these carefully engineered nanostructures hold the promise of new functional materials with enhanced performance. This domain is expected to yield more sophisticated NCs with customizable properties, advancing innovative applications and a deeper understanding of nanoscale materials.

## Supplementary Material



## Notes

Experimental and computational details, X-ray structure analyses, NMR spectra data, luminescence decay curves, computational details. (PDF)

## #

Faculty of Textile Science and Technology, Shinshu University, Tokida, Ueda, Nagano 386-8567, Japan

## Abbreviations

NC: Nanocluster
PL: photoluminescence
dtp: dithiophosphate
CSM: continuous symmetry measure
NPA: natural population analysis
