# Supplementary material for: A Tetrahedral Silver-Rich Supercluster Composed of 8‑Electron IrH2Ag12 Icosahedra
Source: J Am Chem Soc. 2026 May 29;148(22):22481–7. doi: 10.1021/jacs.5c22409 (PMC13266699; doi:10.1021/jacs.5c22409)
Supplement: Supplementary file 1 [file ja5c22409_si_001.pdf]

Supplementary Information

A Tetrahedral Silver-Rich Supercluster Composed of 8-Electron IrH_2Ag_{12} Icosahedra

Tzu-Hao Chiu,[a] Michael N. Pillay,[a] Yoshiki Niihori,[b] Yuichi Negishi,[b] Samia Kahlal,[c] Jean-Yves Saillard,[c] and C. W. Liu[a]*

[a] Department of Chemistry, National Dong Hwa University, Hualien 97401, Taiwan (Republic of China)

[b] Department of Applied Chemistry, Tokyo University of Science, 1-3 Kagurazaka, Shinjuku, Tokyo 162-8601, Japan

[c] Univ Rennes, CNRS, ISCR-UMR 6226, F-35000 Rennes, France

[*] Correspondence: E-mail: chenwei@gms.ndhu.edu.tw; Tel: +886-3-890-3607; Fax : +886-3-890-0162; http://faculty.ndhu.edu.tw/~cwl/index.htm

Experimental Section

1.1 General remarks

All chemicals were purchased from commercial sources and used as received. Solvents were purified following standard protocols. All reactions were carried out under N_2 atmosphere by using standard Schlenk techniques. $[Ag(CH_3CN)_4]PF_6$ and $NH_4[S_2P(O^nPr)_2]$ were prepared by following the procedure reported in literature.[S1,S2] NMR spectra were recorded on a Bruker AVII-400 and AV-600 MHz NMR spectrometers. The chemical shift (δ) is reported in ppm and Hz, respectively. ESI-mass spectra recorded on a Bruker maXis Q-TOF mass spectrometer (Bruker Daltonik GmbH, Germany). UV–visible-NIR absorption spectra were measured on a Shimadzu UV3101PC spectrophotometer at 298 K, using quartz cells with a path length of 1 cm. Luminescence spectra and lifetime were recorded on an Edinburgh FLS920 fluorescence spectrometer. The emission spectra were collected using a HORIBA JOBIN YVON iHR 550 spectrometer and a HORIBA Symphony InGaAs-1700 (for the NIR) detector head were mounted on the exit port. Quantum yields ($\phi_{(em)}$) in 77 K glasses were obtained using $[Ru(bpy)_3]^{2+}$ as the references, and $[Ru(bpy)_3]^{2+}$ in 77K EtOH/MeOH=4:1 glasses were used as references for the determination of emission quantum yields; the quantum yields reported for $[Ru(bpy)_3]^{2+}$ in 77K EtOH/MeOH=4:1 glasses is $\phi_{(em)r} \approx 0.38$,[S3] was used as reference for the determination of relative quantum yields for the complexes studied. eq 1 was used to calculate the relative

quantum yield of target complex ($\phi_{(em)tc}$) in 77 K 2-methyltetrahydrofuran glasses.

$$\frac{\phi_{(em)tc}}{\phi_{(em)r}} = \frac{\eta_{tc}{}^2 I_{tc}}{\eta_r{}^2 I_r} \times \frac{1-10^{-A_r}}{1-10^{-A_{tc}}} \approx \frac{I_{tc}A_r}{I_r A_{tc}}$$

where I_{tc} and I_r are the integrated areas under the emission spectra of target complex (tc, in 2- methyltetrahydrofuran glasses) and reference (r, in 77K EtOH/MeOH=4:1 glasses), respectively, A_{tc} and A_r are the absorbance of interest, respectively, η is refractive index of the solvent, ηtc =1.48 for 2-methyltetrahydrofuran and ηr = 1.45 for EtOH/MeOH. We used cylindrical 2 mm id fluorescence cells immersed in a Dewar with liquid nitrogen for the 77 K emission yield determinations. The sample path length for the absorbance in eq 1, is not well defined for these cells, but the effective path lengths did not vary much since the cell geometry and position were the same for sample and reference solutions.

1.2 Synthesis

1.2.1 Synthesis of [IrH$_2$Ag$_{19}${S$_2$P(O^{n}Pr)$_2$}$_{12}$](1) and [(IrH$_2$)$_4$Ag$_{50}${S$_2$P(O^{n}Pr)$_2$}$_{22}$](2)

In a Flame-dried Schlenk tube, [Ag(CH$_3$CN)$_4$]PF$_6$ (0.50 g, 0.86 mmol) and NH$_4$[S$_2$P(O^{n}Pr)$_2$] (0.14 g, 0.43 mmol) were dissolved in THF, and kept stirring at -20℃ for 5 minutes. [Ir(COD)Cl]$_2$ (0.02 g, 0.043 mmol) was added to the above solution. After 10 minutes, NaBH$_4$ (0.02 g, 0.86 mmol) was added, and kept stirring for 12 hours. The reaction mixture was dried under reduced pressure. The residue was washed with DCM/DI-Water. The DCM layer was dried under reduced pressure and subsequently purified by thin-layer chromatography, with ether:hexane=1:4 as a mobile phase. Finally, yellow brown and gray-black products can be separated.

Yellow brown products [IrH$_2$Ag$_{19}${S$_2$P(O^{n}Pr)$_2$}$_{12}$] **(1)** (Yield: 1% base on Ag)
31P{1H} NMR (161.97 MHz, CDCl3, δ, ppm, r.t.): 105.0 1H NMR (400 MHz, CDCl3, δ, ppm, r.t.): -17.6 (br, H, 2H) 0.95 (t, CH3, 72H), 1.73 (sext, CH2, 48H), 4.11 (q, CH2, 48H). ESI-MS (m/z): exp. 4911.4604 (calc. for [M + Ag]+: 4911.2827) UV-vis (λmax in nm, Ɛ in M-1cm-1): 410 (40000).
Gray-black product [(IrH$_2$)$_4$Ag$_{50}${S$_2$P(O^{n}Pr)$_2$}$_{22}$] **(2)** (Yield: 0.5% base on Ag)
^{31}P{^{1}H} NMR (161.97 MHz, CDCl$_3$, δ, ppm, r.t.): 105.3, 98.7. ^{1}H NMR (400 MHz, CDCl$_3$, δ, ppm, r.t.): -14.0 (br, μ_4-H, 8H) 0.8-1.0 (m, CH$_3$, 132H), 1.67-1.87 (m, CH$_2$, 88H), 4.05-4.36 (m, CH$_2$, 88H). ESI-MS (*m/z*): exp. 5538.7024 (calc. for [M + 2Ag]$^{2+}$: 5538.6659), exp. 5431.8376 (calc. for [M + 2H]$^{2+}$: 5431.7731), exp. 3657.4544 (calc. for [M + Ag + 2H]$^{3+}$: 3657.4838) UV-vis [λ_{max} in nm, (ε in M^{-1}cm^{-1})]: 400 (34000).

1.3 X-ray crystallography

Single crystals suitable for X-ray diffraction analysis of **1** and **2** were obtained by

evaporating MeOH solution at 4°C within a week. The single crystals were mounted on the tip of glass fiber coated in paratone oil, then frozen. Data were collected on a Bruker APEX II CCD diffractometer using graphite monochromated Mo $K\alpha$ radiation ($\lambda = 0.71073$ Å) at 100 K. Absorption corrections for area detector were performed with SADABS[S4] and the integration of raw data frame was performed with SAINT.[S5] The structure was solved by direct methods and refined by least-squares against F^2 using the SHELXL-2018/3 package,[S6,S7] incorporated in SHELXTL/PC V6.14.[S8] All non-hydrogen atoms were refined anisotropically. CCDC 2515650 and 2515651 contain the supplementary crystallo-graphic data for compounds **1** and **2** in this article. These data can be obtained free of charge from the Cambridge Crystallographic Data Centre via www. ccdc.cam.ac.uk/data_request/cif.

1.4 Computational Details

Geometry optimizations were performed by density functional theory (DFT) calculations with the Gaussian 16 package,[S9] using the BP86 functional[S10] and the all-electron Def2-TZVP set from EMSL Basis Set Exchange Library.[S11] All the optimized geometries were characterized as true minima by vibrational analysis. The NAO charges and Wiberg bond indices were computed with the NBO 6.0 program [S12] on single-point calculations performed with the BP86 functional and the Def2-SVP basis set for computational limitations.[S13] The UV-visible transitions were calculated by means of time-dependent DFT (TD-DFT) calculations, with the CAM-B3LYP functional[S14] and the Def2-TZVP basis set. Only singlet-singlet, *i.e.* spin-allowed, transitions have been computed. The UV-visible spectra were simulated from the computed TD-DFT transitions and their oscillator strengths by using the Multiwfn program,[S15] each transition is associated with a Gaussian function of half-height width equal to 2000 cm^{-1}. The compositions of the molecular orbitals were calculated using the AOMix program.[S16]

References

S1. A. A. M. Alyl, B. Walfortn, H. Z. Lang, *Kristallogr. NCS* **2004**, *219*, 489-491.

S2. P. Wystrach, E. O. Hook, G. L. M. Christopher, *J. Org. Chem.* **1956**, *21*, 705-705.

S3. J. N. Demas, G. A. Crosby, *J. Am. Chem. Soc.* **1970**, *92*, 7262.

S4. SADABS, version 2014-11.0, Bruker Area Detector Absorption Corrextions (Bruker AXS Inc., Madison, WI, 2014).

S5. SAINT, included in G. Jogl, V4.043: Software for the CCD detector system (Bruker Analytical: Madison, WI, 1995).

S6. G. M. Sheldrick, *Acta Cryst. A* **2008**, *64*, 112-122.

S7. T. Gruene, H. W. Hahn, A. V. Luebben, F. Meilleur, G. M. J. Sheldrick, *Appl. Cryst.* **2014**, *47*, 462-466.

S8. SHELXTL, version 6.14. (Bruker AXS Inc., Madison, Wisconsin, USA, 2003).

S9. M. J. Frisch, G. W. Trucks, H. B. Schlegel, G. E. Scuseria, M. A. Robb, J. R. Cheeseman, G. Scalmani, V. Barone, G. A. Petersson, H. Nakatsuji, X. Li, M. Caricato, A. V. Marenich, J. Bloino, B. G. Janesko, R. Gomperts, B. Mennucci, H. P. Hratchian, J. V. Ortiz, A. F. Izmaylov, J. L. Sonnenberg, D. Williams-Young, F. Ding, F. Lipparini, F. Egidi, J. Goings, B. Peng, A. Petrone, T. Henderson, D. Ranasinghe, V. G. Zakrzewski, J. Gao, N. Rega, G. Zheng, W. Liang, M. Hada, M. Ehara, K. Toyota, R. Fukuda, J. Hasegawa, M. Ishida, T. Nakajima, Y. Honda, O. Kitao, H. Nakai, T. Vreven, K. Throssell, Jr. J. A. Montgomery, J. E. Peralta, F. Ogliaro, M. J. Bearpark, J. J. Heyd, E. N. Brothers, K. N. Kudin, V. N. Staroverov, T. A. Keith, R. Kobayashi, J. Normand, K. Raghavachari, A. P. Rendell, J. C. Burant, S. S. Iyengar, J. Tomasi, M. Cossi, J. M. Millam, M. Klene, C. Adamo, R. Cammi, J. W. Ochterski, R. L. Martin, K. Morokuma, O. Farkas, J. B. Foresman, D. J. Fox, Gaussian, Inc., Wallingford CT, **2016**; Gaussian 16, Revision A.03.

S10. a) A. D. Becke, *Phys. Rev. A*, **1988**, *38*, 3098-3100; b) J. P. Perdew, *Phys. Rev. B*, **1986**, *33*, 8822-8824.

S11. a) A. Schaefer, H. Horn, R. Ahlrichs, *J. Chem. Phys.* **1992**, *97,* 2571-2577; b) A. Schaefer, C. Huber, R. Ahlrichs, *J. Chem. Phys.* **1994**, *100*, 5829-5835.

S12. a) E. D. Glendening, C. R. Landis, F. Weinhold, *J. Comput. Chem.*, **2013**, *34*, 1429–1437. b) E. D. Glendening, J. K. Badenhoop, A. E. Reed, J. E. Carpenter, J. A. Bohmann, C. M. Morales, C. R. Landis, F. Weinhold, *NBO 6.0*; Theoretical Chemistry Institute, University of Wisconsin, Madison, WI, **2013,** http://nbo6.chem.wisc.edu.

S13. a) F. Weigend, R. Ahlrichs, *Phys. Chem. Chem. Phys*. **2005**, *7*, 3297-3305; b) F. Weigend, *Phys. Chem. Chem. Phys.* **2006**, *8,* 1057-1065.

S14. T. Yanai , D. Tew, N. Handy, *Chem. Phys. Lett.* **2004**, *393*, 51-57.

S15. T. Liu, F. Chen, *J. Comput. Chem*. **2012**, *33*, 580–592.

S16. Gorelsky, S. I. AOMix program, http://www.sg-chem.net.

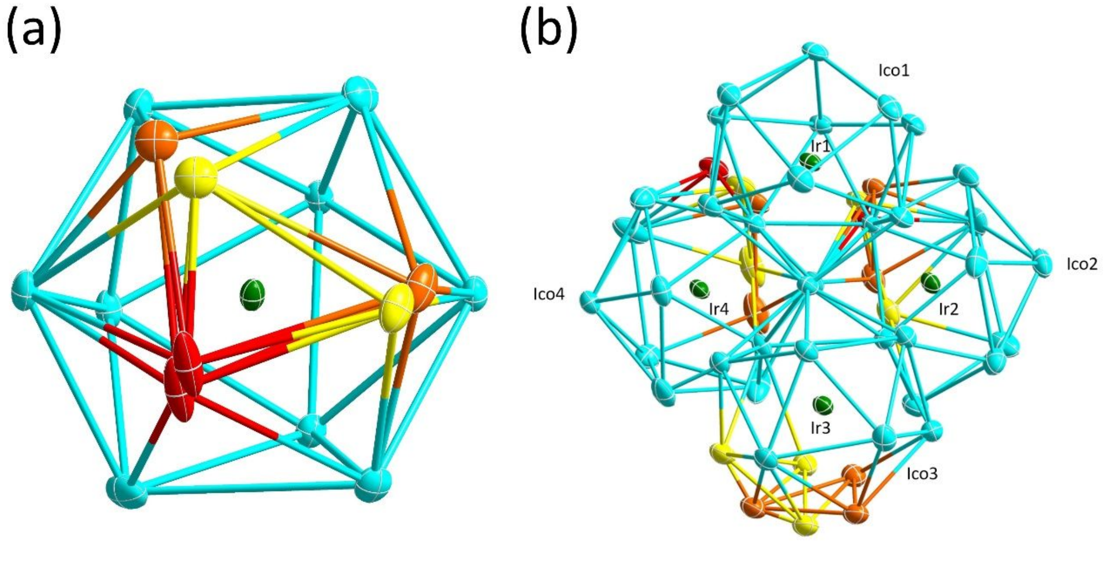

Figure S1. The $[(IrH_2)_4Ag_{42}]^{14+}$ core of **2.**

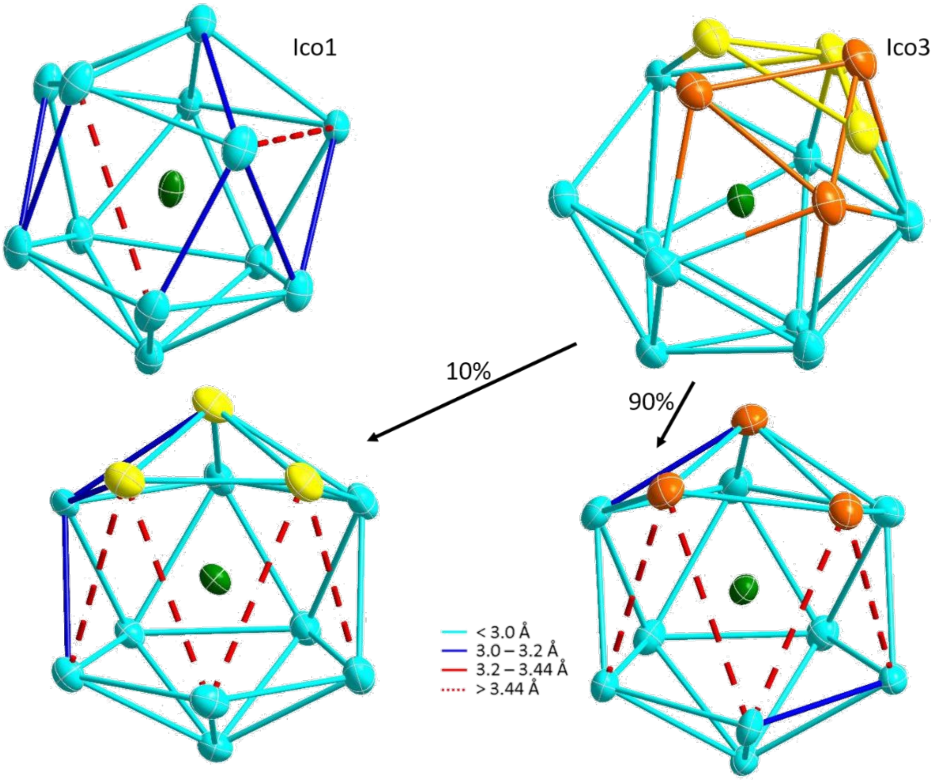

Figure S2. The Ico1 and Ico3 core of **2**.

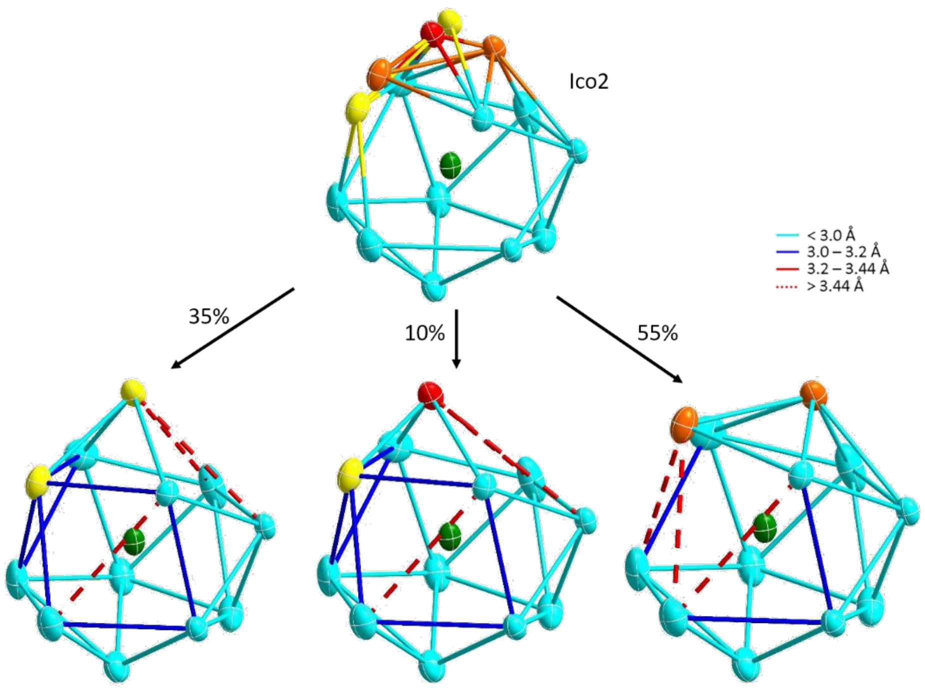

Figure S3. The Ico2 core of **2**.

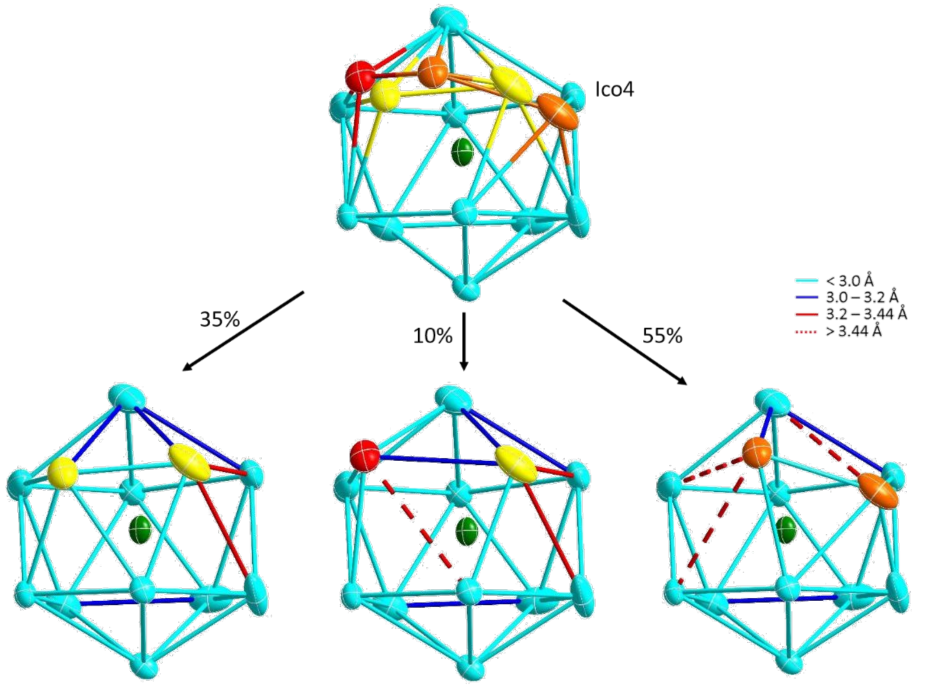

Figure S4. The Ico4 core of **2**.

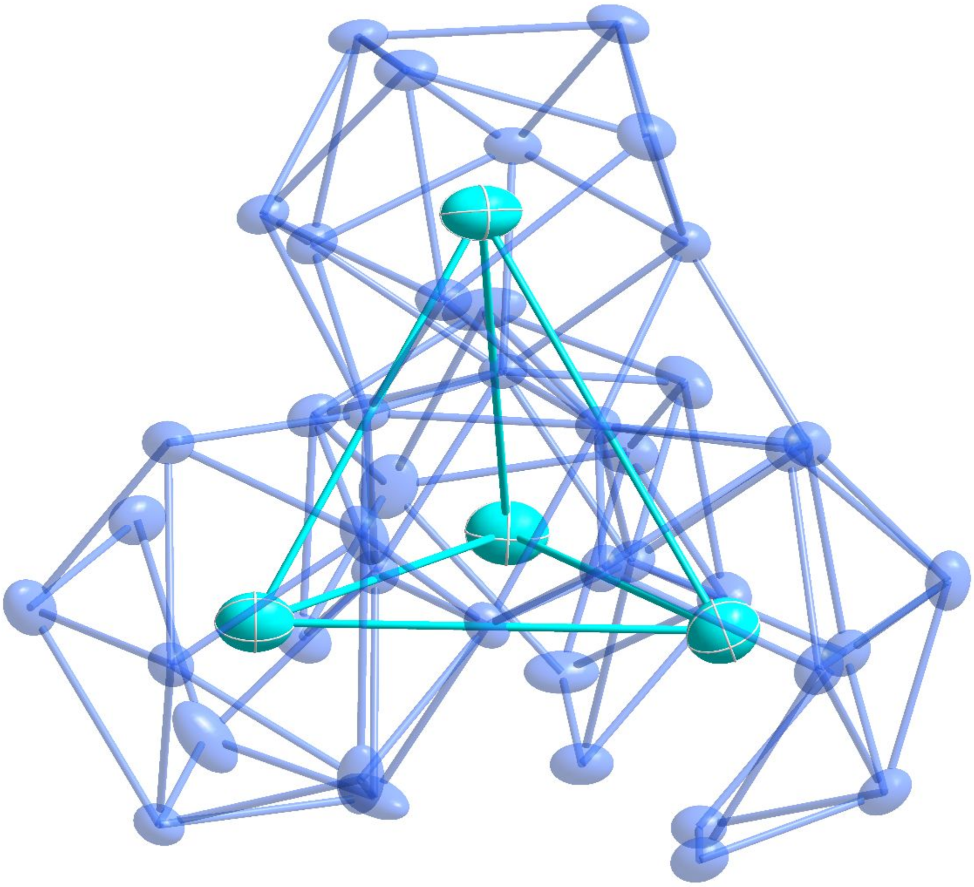

Figure S5. The regular tetrahedron composed of the four Ir.

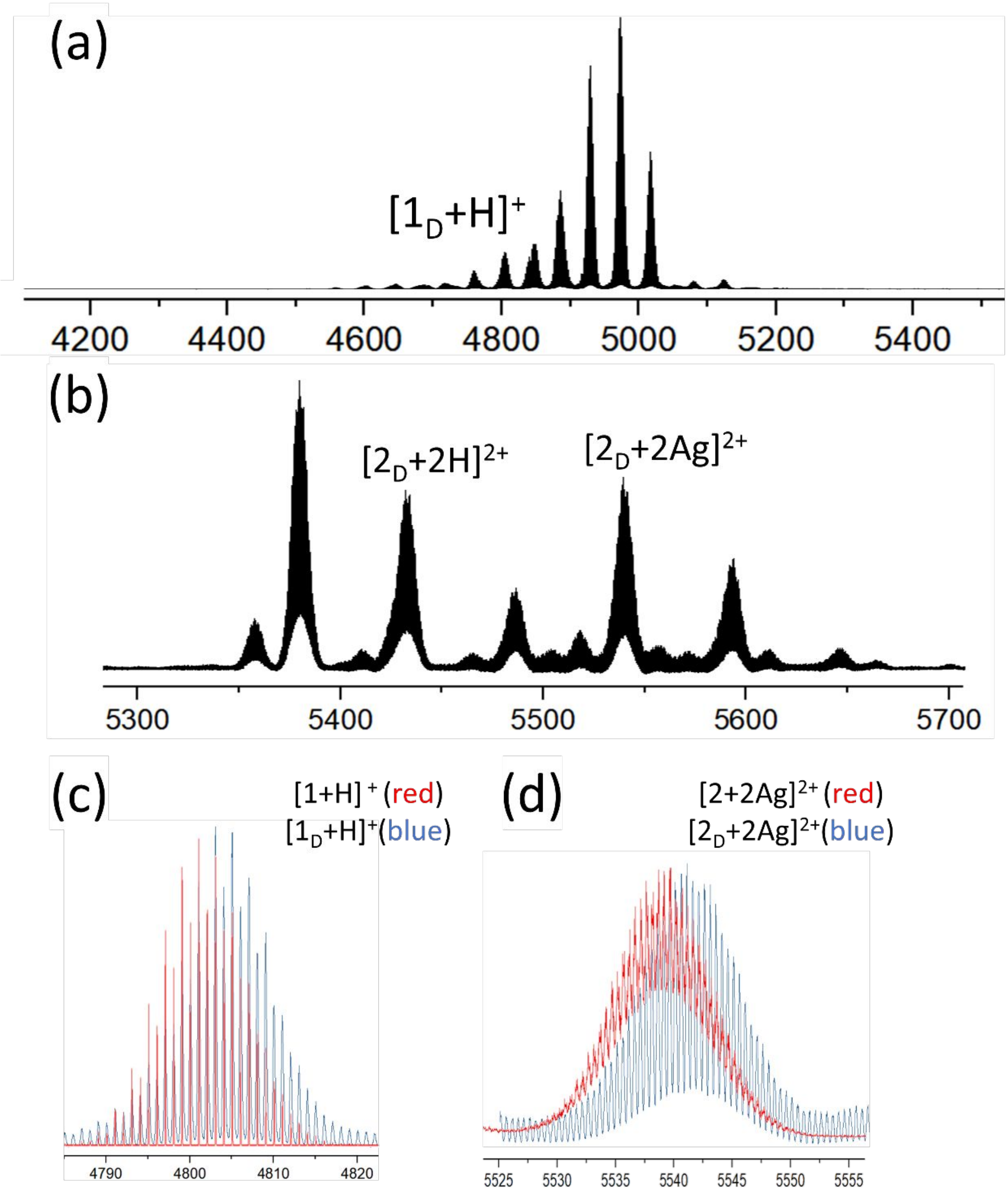

Figure S6. Positive-mode ESI mass spectrum of (a) $\mathbf{1_D}$ (b) $\mathbf{2_D}$ (c) simulated isotopic pattern of [**1**+H]$^+$ and the experimental isotopic pattern of [$\mathbf{1_D}$+H]$^+$ (d) experimental isotopic patterns of [**2**+2Ag]$^{2+}$ and [$\mathbf{2_D}$ + 2Ag]$^{2+}$.

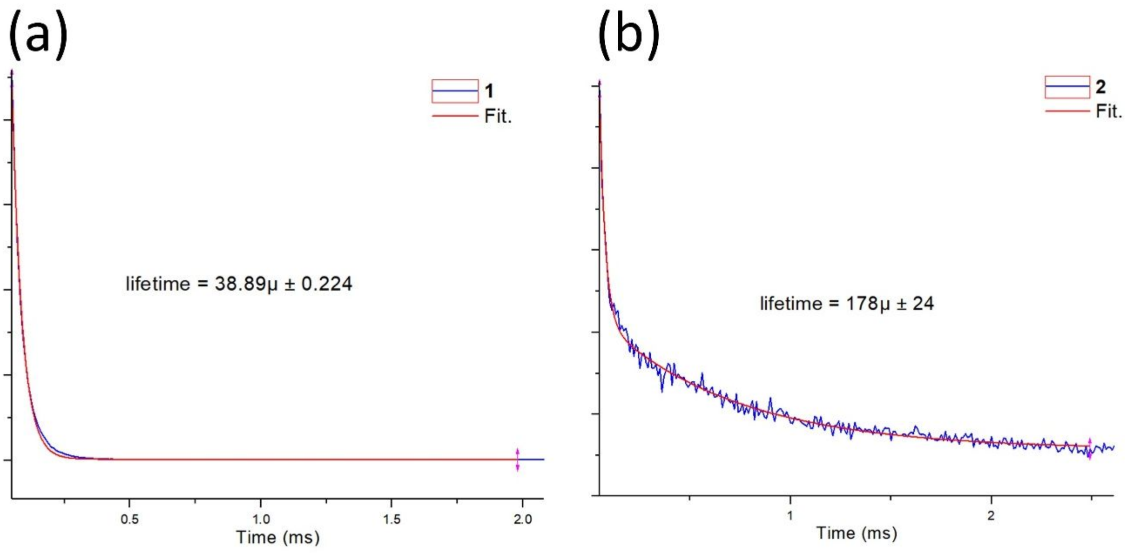

Figure S7. Time-resolved photoluminescence spectrum of **1** and **2**.

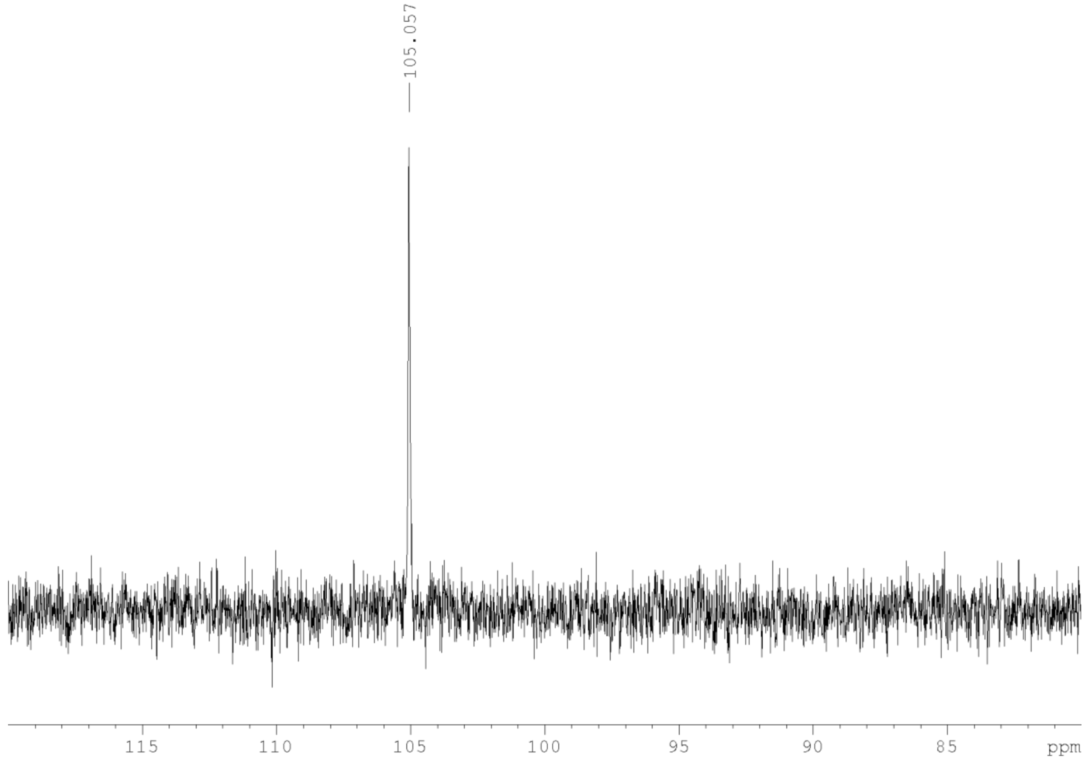

Figure S8. ^{31}P NMR spectrum ($CDCl_3$) of **1**.

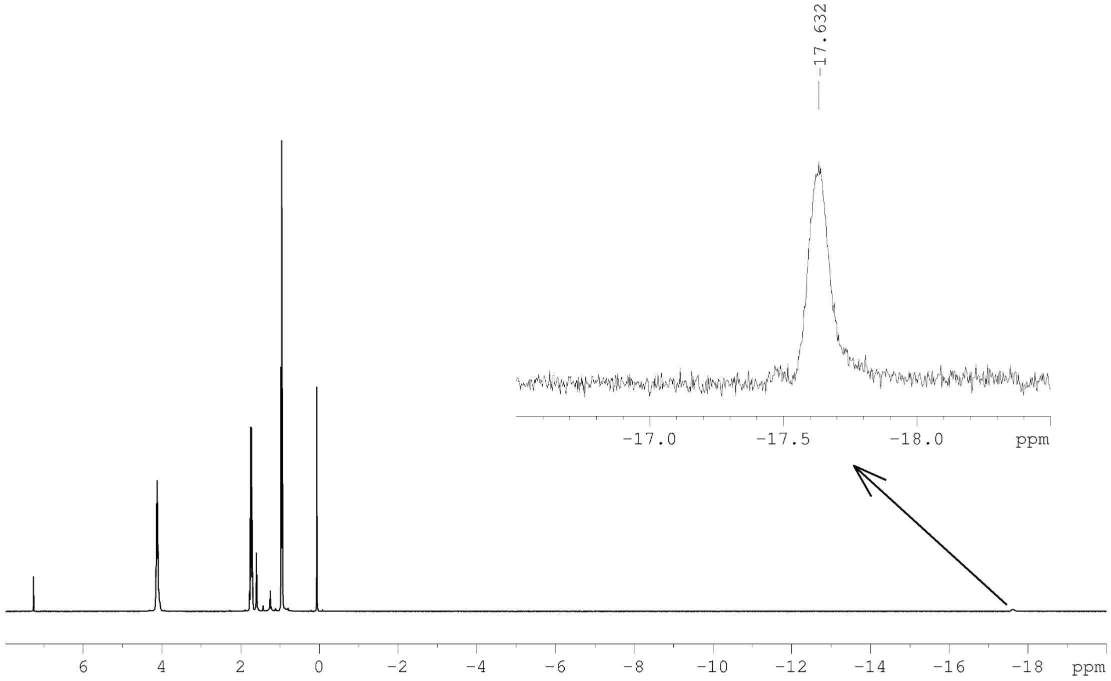

Figure S9. ^{1}H NMR spectrum ($CDCl_3$) of **1**.

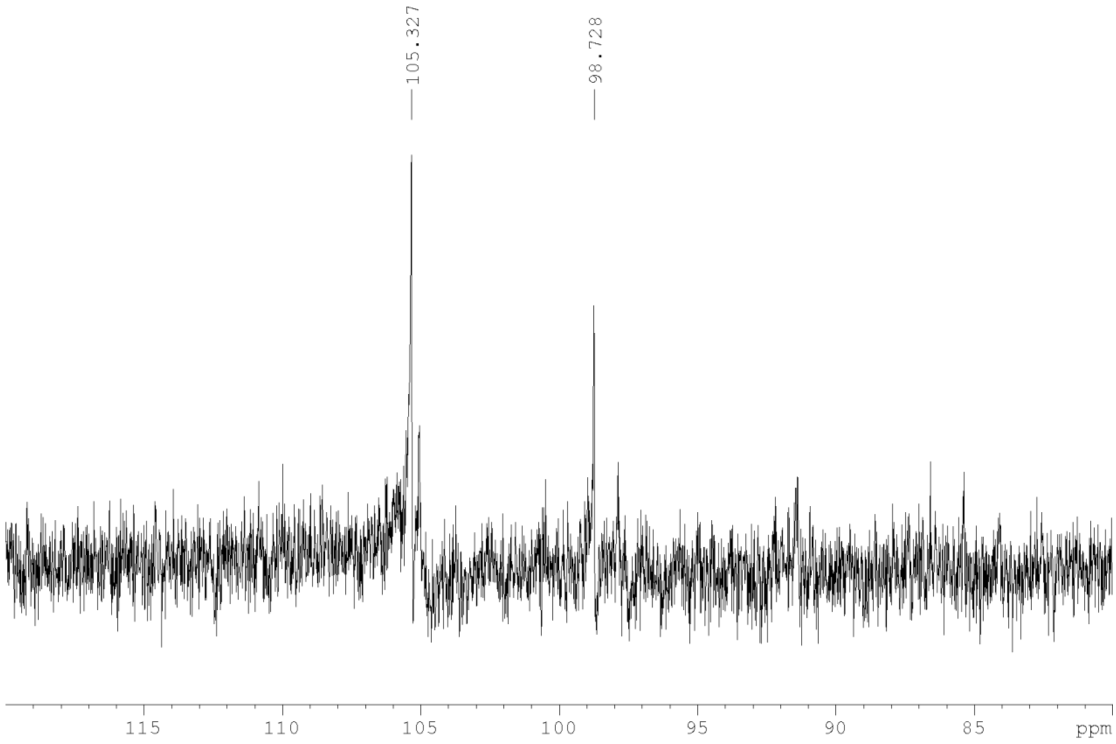

Figure S10. ^{31}P NMR spectrum ($CDCl_3$) of **2**.

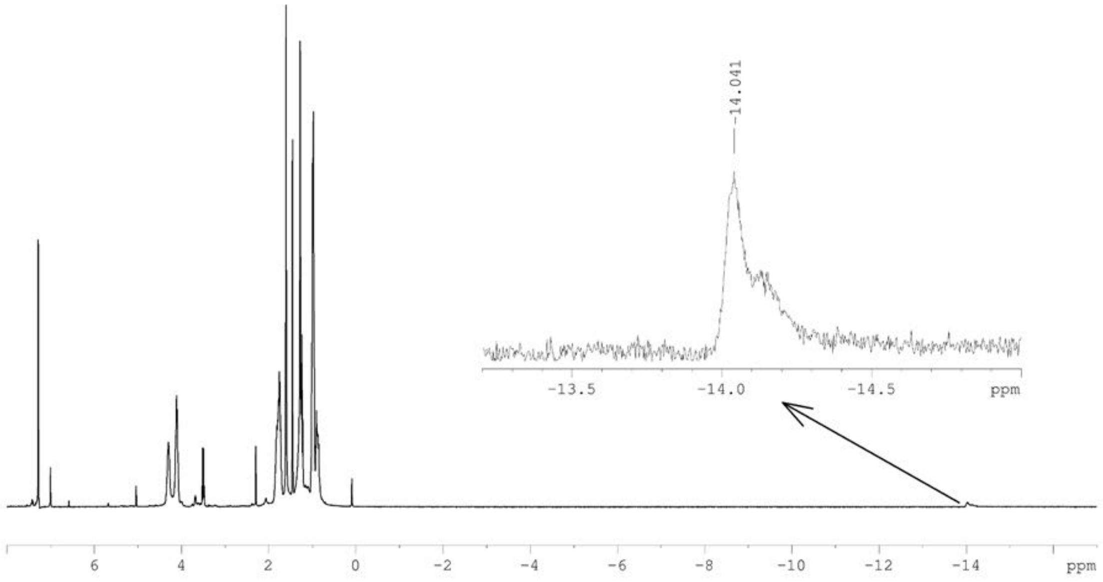

Figure S11. ^{1}H NMR spectrum ($CDCl_3$) of **2**.

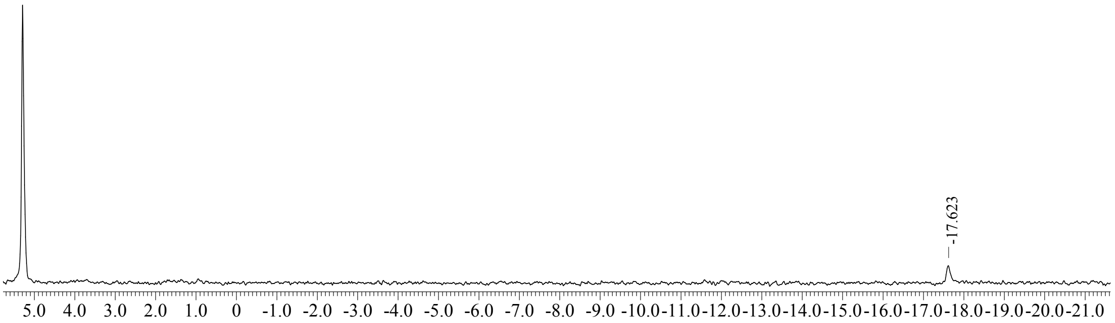

Figure S12. ^{2}H NMR spectrum (DCM) of **1**.

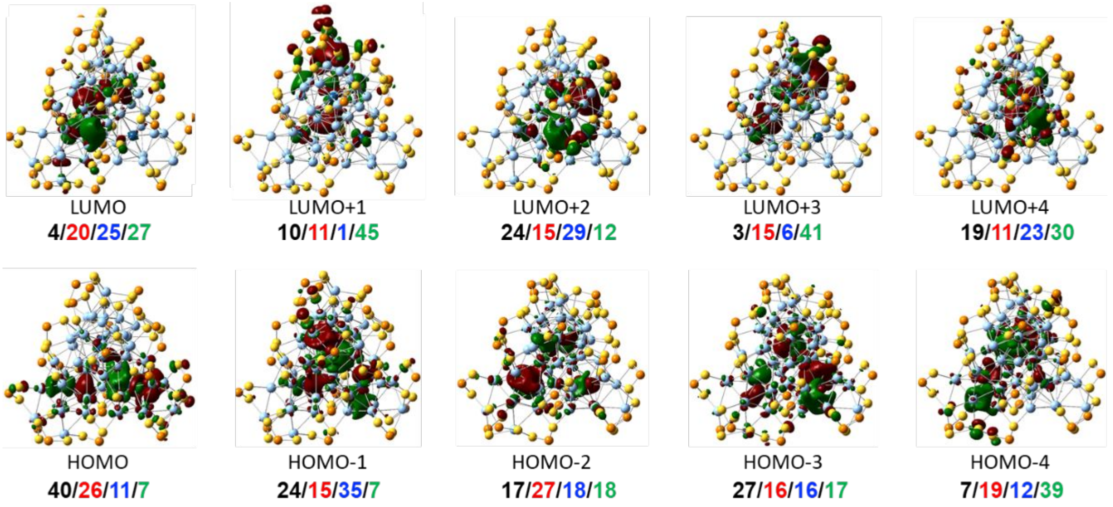

Figure S13. The five highest occupied and five lowest vacant Kohn-Sham orbitals of **2**, with the participation (in %) of each individual icosahedron.

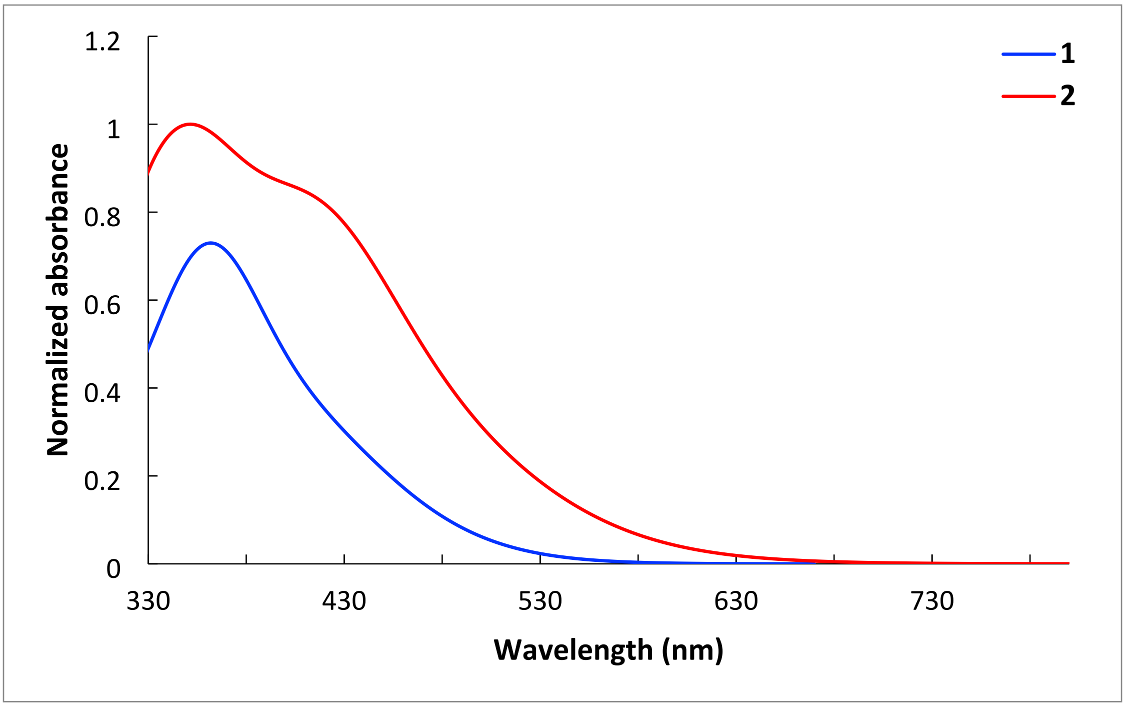

Figure S14. TD-DFT-simulated UV-vis absorption spectra of **1** and **2**. The absorbance units are arbitrary, with the largest calculated value (λ_{max} of **2**) normalized to 1.

Table S1. Selected X-ray crystallographic data of **1** and **2**

Compound	**1**	**2**
CCDC no.	2515650	2515651
Chemical formula	$C_{75}H_{177}Ag_{19}IrO_{24}P_{12}S_{24}$	$C_{132}\ H_{308}\ Ag_{50}\ Ir_{4}\ O_{44}\ P_{22}\ S_{44}$
Formula weight	4845.97	10854.04
Wavelength, Å	0.71073	0.71073
Crystal System	Monoclinic	Monoclinic
Space group	$P2_1/c$	$P2_1/c$
a, Å	30.3647(14)	39.4472(19)
b, Å	17.2806(10)	39.735(2)
c, Å	29.5804(14)	39.235(2)
α, deg.	90	90
β, deg.	110.6123(13)	116.3590(10)
γ, deg.	90	90
V, $Å^3$	14527.8(13)	55105(5)
Z	4	8
Temperature, K	100(2)	100(2)
ρ_{calcd}, g/cm^3	2.216	2.617
μ, mm^{-1}	3.937	5.894
θ_{max}, deg.	25.000	25.000
Completeness, %	99.4	100.0
Reflection collected / unique	79087 / 25448 [R(int) = 0.0479]	329540 / 97078 [R(int) = 0.1203]
Restraints / parameters	553 / 1468	2168 / 5513
$^a R1$, $^b wR2$ [$I > 2\sigma(I)$]	0.0530, 0.1095	0.0735, 0.1538
$^a R1$, $^b wR2$ (all data)	0.0650, 0.1152	0.1452, 0.1963
GOF	1.111	1.027
Largest diff. peak and hole, e/$Å^3$	2.884 and -1.917	3.216 and -2.872

$^a R1 = \Sigma | |F_o| - |Fc| | / \Sigma |F_o|$. $^b wR2 = \{\Sigma[w(F_o^2 - F_c^2)^2]/\Sigma[w(F_o^2)^2]\}^{1/2}$.

Table S2. Cartesian coordinates of the DFT-optimised structures of **1** and **2**.

1

Ir	0.265736	0.184838	0.170536
Ag	2.482719	-1.523239	-0.631506
Ag	2.926997	1.321008	0.329673
Ag	1.294292	0.436833	-2.616267
Ag	-0.182863	-1.940608	-1.776610
Ag	0.177156	-2.615561	1.029163
Ag	2.183229	-0.893176	2.220604
Ag	0.554661	2.575790	1.914877
Ag	-0.816563	-0.355300	2.768493
Ag	-2.297892	-1.192992	0.321389
Ag	-1.651062	0.564112	-1.941891
Ag	-0.146982	2.863594	-0.985013
Ag	-2.095487	1.661476	0.924080
Ag	-2.478428	-3.112389	2.700098
Ag	-1.876149	-4.255773	-0.617616
Ag	-3.300025	-2.021438	-2.421189
Ag	-3.185154	3.251825	-1.507516
Ag	-1.411951	4.802949	1.010293
Ag	4.467470	0.256487	-2.287831
Ag	5.081081	-0.802516	0.965202
H	0.827450	0.591487	1.708943
H	1.160346	1.247381	-0.769486
S	4.879930	0.507013	3.163701
S	3.106947	-2.327292	4.139243
S	4.584669	3.220416	0.060933
S	6.616361	0.448119	-0.778537
S	4.010878	-2.252546	-2.874065
S	4.659698	-3.278689	0.388934
S	3.531037	2.289666	-3.537879
S	1.591045	-0.109440	-5.128114
S	0.392091	5.544962	-0.731340
S	-1.456058	4.103982	-3.299732
S	2.122952	4.393976	2.896193
S	-1.283610	4.431584	3.543109
S	-3.846664	5.220295	0.134152
S	-4.413641	2.206633	1.911070

S	-1.187845	-0.582754	5.271410
S	-4.079474	-1.255044	3.420858
S	-0.288202	-4.199948	3.485608
S	0.356913	-5.303593	0.241544
S	-2.958101	-4.520561	-3.016482
S	0.434827	-3.725288	-3.529579
S	-4.870195	-1.611078	-0.350258
S	-3.859734	-4.733428	1.071743
S	-5.146920	1.667930	-2.022815
S	-2.954090	-0.084078	-4.143802
P	4.576472	-1.005932	4.487235
P	6.367996	2.305741	0.041127
P	5.178284	-3.275680	-1.566269
P	2.831461	1.461243	-5.259213
P	-0.748462	5.768692	-2.397403
P	0.583929	5.126948	3.948879
P	-4.739441	4.165427	1.646528
P	-3.188113	-0.526549	5.093070
P	-0.051055	-5.729203	2.183775
P	-1.328349	-4.322770	-4.248702
P	-5.287731	-3.440609	0.432234
P	-4.650194	1.027559	-3.850352
H	4.405018	-0.378472	5.751467
H	5.799307	-1.696179	4.717275
H	7.288609	3.168348	-0.609670
H	6.927518	2.235870	1.344496
H	5.233075	-4.624583	-2.005854
H	6.537127	-2.888675	-1.722125
H	2.239919	2.527731	-5.990881
H	3.916810	1.144563	-6.123242
H	-1.803558	6.685718	-2.141930
H	0.014787	6.493732	-3.350036
H	0.591558	6.549068	3.900150
H	0.772021	4.927380	5.344111
H	-6.137198	4.374082	1.503840
H	-4.478370	4.900106	2.834369
H	-3.752419	-1.143479	6.243980
H	-3.678736	0.798338	5.243137

H	1.000035	-6.551905	2.666418
H	-1.150579	-6.626942	2.259668
H	-1.168694	-5.584433	-4.880906
H	-1.750730	-3.526059	-5.346360
H	-6.184915	-3.225900	1.509499
H	-6.111261	-4.142188	-0.486635
H	-5.739310	0.293152	-4.390021
H	-4.553718	2.092123	-4.785694

2

Ir	-0.838111	0.197286	3.607537
Ir	-2.955821	-1.476006	-1.806364
Ir	0.545783	3.158725	-1.498451
Ir	2.737649	-2.251954	-0.558050
Ag	-1.632881	-1.439738	5.728249
Ag	0.756778	0.454349	6.264157
Ag	-3.068404	1.362497	5.291119
Ag	-3.537393	-0.780425	3.421681
Ag	-1.329816	-2.607171	3.055667
Ag	1.099292	-1.793762	4.463725
Ag	1.866913	0.929182	3.579979
Ag	-0.497661	2.965580	4.759147
Ag	-2.743820	1.910115	2.435156
Ag	-1.956171	-0.591400	0.944095
Ag	0.849290	-1.070199	1.639431
Ag	0.027318	1.745506	1.111785
Ag	-4.141268	0.845222	-0.328604
Ag	-4.666646	-1.857152	0.566105
Ag	-5.835574	-1.014140	-1.953493
Ag	-4.565510	-3.784108	-1.733189
Ag	-2.277702	-3.466687	0.218902
Ag	-0.092034	-1.896495	-1.089196
Ag	-1.414917	0.974468	-1.477801
Ag	-3.843881	0.859119	-3.178417
Ag	-4.431081	-1.740295	-4.407232
Ag	-1.639030	-3.929485	-2.547712
Ag	-1.683010	-0.607616	-4.454340
Ag	-1.997669	3.486382	-0.114228
Ag	-1.850535	3.481333	-3.014959

Ag	0.403571	2.085457	-4.285036
Ag	2.750277	2.920265	0.342264
Ag	0.486886	4.643125	0.978131
Ag	-0.788589	5.794044	-1.413193
Ag	0.594729	4.938517	-3.752886
Ag	3.045750	3.477845	-3.407728
Ag	2.215770	5.454021	-1.241451
Ag	1.649630	0.447828	-0.839472
Ag	2.186834	-1.130899	-3.189413
Ag	3.613201	-0.087089	1.238707
Ag	1.445500	-3.828763	-2.734399
Ag	0.854624	-4.562124	0.102974
Ag	3.307098	-2.875974	2.174423
Ag	4.135695	-4.762311	0.077044
Ag	4.500160	-2.948366	-2.635517
Ag	4.399207	-0.132232	-1.633059
Ag	5.468802	-2.118627	0.212259
H	-1.056154	0.769728	5.163307
H	-0.345187	1.727803	3.122390
H	1.644752	-2.679602	0.623274
H	2.752083	-3.838461	-1.118088
H	2.149948	2.821331	-1.732187
H	0.470906	1.651383	-2.178372
H	-1.474489	-1.016984	-2.404286
H	-3.018035	-2.468674	-3.156302
Ag	-1.571281	1.419498	8.061361
Ag	-7.252681	-3.334495	-0.318663
Ag	-7.073108	-3.183129	-3.678690
Ag	0.797233	7.683378	0.446388
Ag	1.731315	7.780130	-3.058566
Ag	7.186824	-1.444973	-2.148126
Ag	6.861393	-4.484766	-1.224395
Ag	6.070935	-4.246515	2.350872
S	0.456041	5.160184	3.515574
S	-1.411149	7.565198	1.780690
S	-2.214157	7.652454	-2.509166
S	0.618804	9.515636	-1.488599
S	1.050695	6.890699	-5.400011

S	4.140841	5.105475	-5.128170
S	3.199383	7.520550	1.363585
S	4.089708	7.146383	-2.024238
S	5.209616	2.096391	-2.611258
S	7.679093	0.161822	-4.126449
S	4.430235	0.991838	3.506309
S	5.127236	3.414150	1.077501
S	7.838910	-0.921076	0.409879
S	8.370034	-4.389291	0.987901
S	8.418053	-3.623315	-3.138899
S	5.307877	-4.260738	-4.675446
S	5.585893	-6.776008	-1.328249
S	4.834695	-6.581847	2.060327
S	6.541788	-3.170398	4.649333
S	3.065103	-3.503857	4.663325
S	0.444687	-7.011901	-0.736147
S	-0.141007	-5.511085	-3.909498
S	0.826689	-5.299579	2.664757
S	-2.630765	-4.829170	2.414665
S	-5.170780	-6.271132	-1.711952
S	-8.383880	-4.869174	-2.167220
S	-5.464872	-0.980834	-6.678675
S	-5.719289	-4.262752	-5.598472
S	-2.210487	-2.470313	7.965858
S	0.359228	-0.154080	8.837113
S	-6.295683	-4.598595	1.723008
S	-5.986062	-1.297046	2.819497
S	-8.034720	-0.748316	-3.642860
S	-8.357238	-0.982727	-0.179280
S	-4.055069	0.860750	8.087853
S	-4.478529	3.500711	5.876777
S	-6.192320	2.098021	0.566064
S	-3.626679	4.246564	1.738915
S	-0.745437	3.786478	7.317559
S	2.468760	2.533614	6.592058
S	-1.396055	1.409142	-6.199365
S	-3.998072	3.123253	-4.453564
S	2.259473	0.422080	-5.306746

S	0.082354	-2.299583	-5.495396
P	-0.925149	6.668431	3.509597
P	-1.385320	9.356779	-1.875590
P	2.739088	6.039271	-6.207992
P	4.193593	8.169918	-0.252845
P	6.099871	1.380232	-4.308661
P	4.978804	2.906360	3.003696
P	8.999384	-2.460335	1.030363
P	7.233935	-3.712140	-4.802069
P	5.833468	-7.489068	0.550439
P	4.762471	-2.540614	5.312011
P	-0.123107	-7.136623	-2.647002
P	-1.026072	-6.059215	2.698054
P	-7.049462	-6.401011	-2.395466
P	-5.957762	-2.887010	-7.068806
P	-0.502859	-2.014481	8.907379
P	-6.439997	-3.254872	3.204195
P	-9.221885	-0.812627	-1.991654
P	-4.920764	2.647529	7.637220
P	-5.525755	3.922080	1.037319
P	1.274004	3.735375	7.661496
P	-3.118634	2.477610	-6.173273
P	1.337573	-1.029525	-6.411567
H	-2.116677	6.200849	4.122619
H	-0.482564	7.618759	4.469263
H	-2.026026	9.841214	-0.703933
H	-1.701248	10.385610	-2.802268
H	2.246365	5.209531	-7.250471
H	3.377702	7.059520	-6.961576
H	5.574031	8.251350	0.068790
H	3.903848	9.535531	-0.522208
H	5.088485	0.810673	-5.129733
H	6.477444	2.512918	-5.076210
H	4.144898	3.785741	3.743058
H	6.233953	3.115421	3.632012
H	9.402125	-2.167323	2.358319
H	10.238288	-2.409216	0.339487
H	7.369977	-2.460313	-5.460522

H	7.880589	-4.591323	-5.710144
H	7.217051	-7.582990	0.862849
H	5.469264	-8.861671	0.551357
H	4.742401	-2.614330	6.729942
H	4.590965	-1.143263	5.107762
H	-1.414226	-7.716672	-2.760353
H	0.665481	-8.106782	-3.321066
H	-1.240591	-6.703212	3.944967
H	-1.148144	-7.142159	1.787334
H	-7.653318	-7.551131	-1.821114
H	-7.074155	-6.743710	-3.774118
H	-7.283798	-2.937580	-7.581230
H	-5.237425	-3.376556	-8.192709
H	-0.665377	-2.318692	10.284812
H	0.534082	-2.919950	8.556664
H	-5.671767	-3.709694	4.308088
H	-7.740730	-3.230501	3.776681
H	-10.011108	0.367799	-1.998097
H	-10.225475	-1.802861	-2.174019
H	-6.323039	2.446346	7.761994
H	-4.710070	3.570690	8.699305
H	-6.385833	4.492688	2.011695
H	-5.696893	4.836132	-0.037121
H	1.451730	3.525450	9.055826
H	1.731781	5.073825	7.535089
H	-4.093246	1.749288	-6.903521
H	-2.923591	3.618838	-6.993440
H	0.710384	-0.407909	-7.524507
H	2.365391	-1.774404	-7.045161
